# Highest Vaccine Uptake after School-Based Delivery - A County-Level Evaluation of the Implementation Strategies for HPV Catch-Up Vaccination in Sweden

**DOI:** 10.1371/journal.pone.0149857

**Published:** 2016-03-14

**Authors:** Moa Rehn, Ingrid Uhnoo, Sharon Kühlmann-Berenzon, Anders Wallensten, Pär Sparén, Eva Netterlid

**Affiliations:** 1 Department of Monitoring and Evaluation, Public Health Agency of Sweden, Solna, Sweden; 2 European Programme for Intervention Epidemiology Training (EPIET), European Centre for Disease Prevention and Control (ECDC), Stockholm, Sweden; 3 Section for Infectious Diseases, Department of Medical Sciences, Uppsala University, Uppsala, Sweden; 4 Department of Medical Epidemiology and Biostatistics, Karolinska Institutet, Solna, Sweden; 5 Department of Clinical Sciences, Lund University, Malmö, Sweden; 6 Department of Occupational and Environmental Dermatology Skåne University Hospital, Lund University, Malmö, Sweden; Rudjer Boskovic Institute, CROATIA

## Abstract

**Background:**

The Swedish school-based vaccination programme offers HPV vaccine to girls born ≥1999 in 5-6th grade. In 2012, all counties introduced free-of-charge catch-up vaccination campaigns targeting girls born 1993–1998. Varying vaccine uptake in the catch-up group by December 2012 suggested that some implementation strategies were more successful than others. In order to inform future vaccination campaigns, we assessed the impact of different implementation strategies on the county-level catch-up vaccine uptake.

**Methods:**

We conducted an ecological study including all Swedish counties (n = 21), asking regional health offices about the information channels they used and where vaccination of the catch-up target group took place in their counties. The uptake of ≥1 dose by 30 September 2014 was estimated using data from the voluntary national vaccination register. We investigated associations between counties’ catch-up vaccine uptake, information channels and vaccination settings by calculating incidence rate ratios (IRR) and 95% confidence intervals (CI), using negative binomial regression models.

**Results:**

County level catch-up vaccine uptake varied between 49–84%. All counties offered vaccination through primary health care settings. Apart from this eight (34%) also offered the vaccine in some of their schools, four (19%) in all their schools, and two (10%) in other health care centres. The information channels most frequently used were: information at the national on-line health care consulting web-page (100%), letter/invitations (90%), and advertisement (81%). Counties offering vaccination to girls in all schools and counties offering vaccination in some of their schools, reached higher vaccine uptake compared to counties not offering vaccination in any of their schools (all schools adjusted IRR: 1.3, 95% CI: 1.1–1.5, some schools adjusted IRR: 1.2, 95% CI: 1.1–1.3).

**Conclusion:**

Counties offering HPV vaccination to catch-up groups in schools reached the highest vaccine uptake. No information channel explained differences in county-level vaccine uptake. Our findings suggest that catch-up vaccination outside the national vaccination program can reach a high uptake at the population level if it is implemented primarily with an organized delivery (e.g. in schools).

## Introduction

Cervical cancer causes 530 000 cases and 275 000 deaths among women worldwide each year [1]. In the Nordic countries it has been estimated that half of the expected cervical cancer cases may have been prevented by the introduction of screening in the late 1960s and early 1970s [2]. In 2013, 466 new cases were recorded in Sweden, less than half the incidence recorded in the 1960s [3].

Infection with Human papilloma virus (HPV) is the main cause of developing cervical cancer [4]. Two vaccines protecting against the two HPV genotypes (16 and 18) responsible for nearly 70% of cervical cancers are currently available in Sweden [5].

Estimations based on 179 countries show that vaccination of 12 year-old girls (total cohort of 58 million girls) would prevent 690 000 cases of cervical cancer, and among those, 420 000 deaths [6]. Since 2009, the World Health Organization has recommended inclusion of the HPV vaccine in the national immunization programs [7]. A review of the first five years of HPV vaccine introduction [8] concluded that in 2012, at least 39 countries had implemented HPV vaccination programs, and that countries with publicly financed school-based vaccinations reached higher vaccination coverage compared to other implementation strategies. HPV vaccination coverage in school-based vaccination programs have been reported to be especially successful among younger girls in the primary target group [9], and in general parents tend to support the vaccination to be delivered to adolescents through school [10]. However, Denmark reached high coverage through a publicly financed general practitioner based delivery model where girls where personally invited [11, 12], suggesting that an organized delivery is effective in general and not only when organized through schools.

In 2010, the HPV vaccine was included in the Swedish free-of-charge national vaccination program targeting all girls born 1999 or later and attending the 5^th^ or 6^th^ grade in school [13, 14]. However, the vaccinations did not start until 2012 due to delays in the procurement process. At the same time, all counties additionally introduced free-of-charge catch-up vaccinations targeting girls born 1993–1998. The quadrivalent vaccine, targeting the oncogenic HPV16 and HPV18 and the genital wart responsible HPV6 and HPV11, is used in both programs.

The primary target group for vaccination is girls between 10 and 12 years old, initially administered in a three-dose schedule, with the second and third doses recommended at 2 and 6 months after the first dose [14]. Since 2015 however, a two-dose schedule is recommended for girls up to and including 13 years of age, whereas the three-dose schedule still applies to girls 14 years old and above [15].

HPV vaccination within the national vaccination program is delivered through the local school health care services, whereas catch-up vaccination is organized at county level with a variety of different implementation strategies. In December 2012, the varying proportion of the catch-up group initiating the HPV vaccine series (henceforth referred to as vaccine uptake (%)) suggested that some counties were more successful than others. At that point, some counties made an effort to increase the vaccine uptake by applying changes in their strategies. In our study we assessed the impact of the implementation strategies for HPV vaccination on county level vaccine uptake in the catch-up group, as well as the effect of changing the strategy after the first year, in order to inform future campaigns.

## Methods

We performed an ecological study aimed at calculating the HPV vaccine uptake in 2012 and 2014 among the catch-up target population which included 325 229 girls born between 1993 and 1998 living in Sweden.

### Ethics statement

The study was reviewed and approved by the Stockholm regional research ethics review board (registration number: 632/2006-76 and 67/2014-341). Informed consent was obtained from study participants.

### Data collection

#### Vaccine uptake

In 2006 the Public Health Agency of Sweden started to monitor HPV vaccination through a voluntary vaccination registry, and since 2013 mandatory registration of all vaccinations included in the national vaccination programme is required by Swedish law [16]. The catch-up vaccinations, however, are not part of the national vaccination programme and therefore not mandatory to register. Therefore, all HPV vaccinations analysed in this study have been registered by the vaccinator in a voluntary reporting system. Registration was done using an opt-out principle, i.e. consent to registration was assumed if the girl (or her parent) did not oppose registration after being informed about this. Compared to the number of vaccine doses sold nationally, between 85–90 percent of all administered doses were registered in the voluntary vaccination registry.

We aggregated the number of girls born 1993–1998 who received at least one HPV vaccine dose by 31 December 2012 and by 30 September 2014; this was done by county (n = 21). The number of girls born 1993–1998, i.e. the catch-up target population, was obtained from Statistic Sweden (SCB) [17] for 2011 and 2013 and by county; these data were used as denominators when calculating the county-level vaccine uptake (%) in 2012 and 2014 respectively. We also aggregated by county the number of vaccinated girls born 1999–2001 (primary target group) by 30 September 2014.

#### Implementation strategies

**Fig 1:**
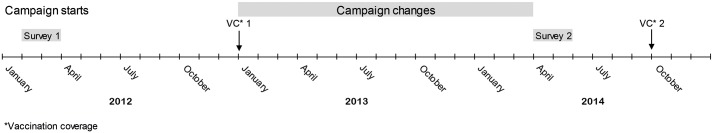
Timeline. Schematic description of the activities carried out during the study period.

We collected information about implementation strategies for catch-up vaccination from all 21 county health care offices at two points in time (Fig 1), using two different data collection tools. The first was at the beginning of the campaign in February-March 2012 when we e-mailed an open ended questionnaire asking representatives to list and describe the information channels used to reach the target group, and the settings where the girls were offered the vaccine. The second occasion took place in April-May 2014 when we contacted the representatives by telephone to conduct structured open ended interviews, asking them to list and describe if they added information channels and settings after 2012.

### Data analysis

The information channels and vaccination settings that were listed by the county health care offices is described in Table 1. Information channels were dichotomized for each county as yes/no meaning it was used or not by the county. The vaccination settings were primary health care centres (yes/no), schools (categorized as vaccination in all schools, some schools, or no schools) and other health care centre (yes/no) (Table 1).

**Table 1:** Description of information channels and vaccination settings used among counties to implement catch-up vaccination of girls born 1993–1998, Sweden 2012–2014.

Variable	Description
*Information channel*	
Targeted information	The county organized oral and/or written information to non-native Swedish speakers in other languages.
Smart phone app	The county launched an application for smartphones, free of charge. The application provided written and visual information and reminders about next dose.
County website	The county provided written information on a specific page at the counties’ official webpage.
Media coverage	Information through media using press releases, interviews with health professionals in the radio, TV, and newspapers (on- and off-line).
School-based information	Information provided through the established information channels in each school (e.g. e-mail, information letters, meetings for parents, oral information from the school nurse).
Letter/invitation	Written information provided through a letter/invitation to girls and/or the parents including consent form.
Advertisement	Information through advertisement in newspapers (on- and off-line), posters, booklets.
Social media	Written information from the county health care office in social media.
Cinema commercial/YouTube	Visual information in a film available on YouTube and/or in local cinemas.
On-line health care consulting	Written information on the county-specific page on a nationwide health care consulting web site (*Vårdguiden 1177*).
*Vaccination setting*	
All schools	Organized delivery of the vaccine in all schools in the county. Either through the established school health care or through nurses touring around to all schools in the county to administer the vaccine.
Some schools	Organized delivery of the vaccine in schools in some of the municipalities in the county, or to selected birth cohorts. Either through the established school health care or through nurses touring around these schools to administer the vaccine.
Other health care centre	Opportunistic delivery of the vaccine at private vaccination centres or midwife clinics.
Primary health care centre	Opportunistic delivery of the vaccine at the primary health care centres across the county.

The county-level catch-up vaccine uptake by 31 December 2012 and 30 September 2014 was calculated for the catch-up group (born 1993–1998) by county (n = 21). The vaccine uptake was further stratified in two sub-groups in order to study the differences between those attending compulsory lower secondary school (7^th^ − 9^th^ grade) and those attending voluntary upper secondary school (10^th^-12^th^ grade) at the start of the catch-up campaign in 2012, i.e. girls born 1996–1998 and girls born 1993–1995.

We then investigated associations between counties’ catch-up vaccine uptake, and information channels and vaccination settings. Negative binomial regression models with number of vaccinated as outcome and catch-up target population as offset were used to calculate incidence rate ratios (IRR) and 95% confidence intervals (CI) at county level; negative binomial regression was used to compensate for over dispersion in the outcome. We first calculated crude IRR in univariate models for each information channel and vaccination setting listed in Table 1. We then included all variables as well as vaccine uptake in the primary target group in a multivariable model in order to obtain adjusted IRRs. Vaccine uptake in the primary target group was included to adjust for possible county variations in uptake of vaccines in the national vaccination program. The analyses were performed on the vaccine uptake for the whole catch-up group, and also separately for each of the two strata described above.

To investigate if the change in vaccine uptake between 31 December 2012 and 30 September 2014 was associated with the introduction of additional information channels and/or settings after 31 December 2012, we fitted a negative binomial regression model with the additional number of vaccinated girls after 31 December 2012 as outcome and catch-up target population as offset. The independent variable was introduction of additional channels and/or settings (yes/no). We adjusted the analysis for the vaccine uptake in 2012 (i.e. the vaccine uptake before changing the strategy).

All analyses were performed using Stata 13. We considered CI excluding 1.0 as statistically significant.

## Results

All 21 county health care offices replied to each of the surveys. Ten information channels and four vaccination settings were included in the analysis (Table 1 & S1 Table). All counties offered vaccination to the catch-up group through primary health care settings, eight (34%) additionally offered the vaccine in some of their schools, four (19%) in all their schools, and two (10%) in other health care centres. Counties used between one to eight information channels throughout the campaign. The information channels most frequently used were: information at on-line health care consulting (100%), letter/invitations to individuals (90%), advertisement (81%), school-based information (48%) and county websites (43%) (Table 2).

**Table 2:** Impact of implementation strategy on catch-up vaccine uptake among girls born 1993–98 and stratified by two age-groups, Sweden 2012–2014.

	Counties	Girls born 1993–1998		Voluntary education (born 1993–95)		Compulsory education (born 1996–98)	
Explaining factors	implemented	IRR (95% CI)		IRR (95% CI)		IRR (95% CI)	
	n (%)	Crude	Adjusted^b^	Crude	Adjusted^b^	Crude	Adjusted^b^
*Information channel*^a^							
Smart phone app	3 (14)	0.9 (0.7–1.0)	1.1 (1.0–1.2)	0.8 (0.7–1.0)	1.0 (0.9–1.1)	0.9 (0.7–1.1)	1.2 (1.0–1.4)
Media coverage	8 (38)	1.0 (0.8–1.1)	1.1 (1.0–1.2)	1.0 (0.8–1.1)	1.0 (1.0–1.1)	1.0 (0.8–1.2)	1.1 (1.0–1.2)
County website	9 (43)	1.0 (0.9–1.1)	1.1 (1.0–1.1)	1.0 (0.8–1.1)	1.0 (1.0–1.1)	1.0 (0.9–1.2)	1.1 (1.0–1.2)
School—based information	10 (48)	1.2 (1.0–1.3)	1.0 (1.0–1.1)	**1.2 (1.1–1.3)**	1.1 (1.0–1.1)	1.1 (1.0–1.3)	1.0 (0.9–1.1)
Social media	6 (29)	0.9 (0.8–1.1)	1.0 (0.9–1.0)	0.9 (0.8–1.1)	0.9 (0.9–1.0)	1.0 (0.8–1.1)	1.0 (0.9–1.1)
Targeted information	2 (10)	0.9 (0.7–1.1)	0.9 (0.8–1.1)	0.9 (0.7–1.1)	0.9 (0.8–1.0)	0.9 (0.7–1.2)	1.0 (0.8–1.2)
Advertisement	17 (81)	0.9 (0.7–1.0)	0.9 (0.9–1.0)	0.8 (0.7–1.0)	0.9 (0.9–1.0)	0.9 (0.8–1.1)	0.9 (0.8–1.0)
Letter/invitation	19 (90)	**0.7 (0.6–0.9)**	0.9 (0.8–1.0)	**0.7 (0.6–0.8)**	0.9 (0.8–1.0)	**0.7 (0.6–0.9)**	0.9 (0.8–1.1)
Cinema commercial/YouTube	8 (38)	0.9 (0.8–1.0)	0.9 (0.8–1.0)	0.9 (0.8–1.0)	1.0 (0.9–1.1)	0.9 (0.8–1.0)	0.9 (0.8–1.0)
*Vaccination setting*							
No schools (ref)		**1.0**		**1.0**	**1.0**	**1.0**	**1.0**
All schools	4 (19)	**1.4 (1.2–1–5)**	**1.3 (1.1–1.5)**	**1.3 (1.2–1.5)**	**1.2 (1.1–1.4)**	**1.4 (1.2–1.6)**	**1.4 (1.1–1.6)**
Some schools	8 (38)	1.0 (0.9–1.1)	**1.2 (1.1–1.3)**	1.0 (0.9–1.1)	**1.2 (1.1–1.3)**	1.0 (0.9–1.2)	**1.3 (1.2–1.4)**
Other health care centre^a^	2 (10)	0.9 (0.7–1.1)	0.9 (0.8–1.0)	0.9 (0.7–1.1)	0.9 (0.8–1.0)	0.9 (0.7–1.1)	0.8 (0.7–1.0)

^a^ counties not implementing the explaining factor used as reference group

^b^ multivariable model including all explaining factors as well as vaccine uptake in national vaccination program

Bold indicates statistically significant associations

**Fig 2:**
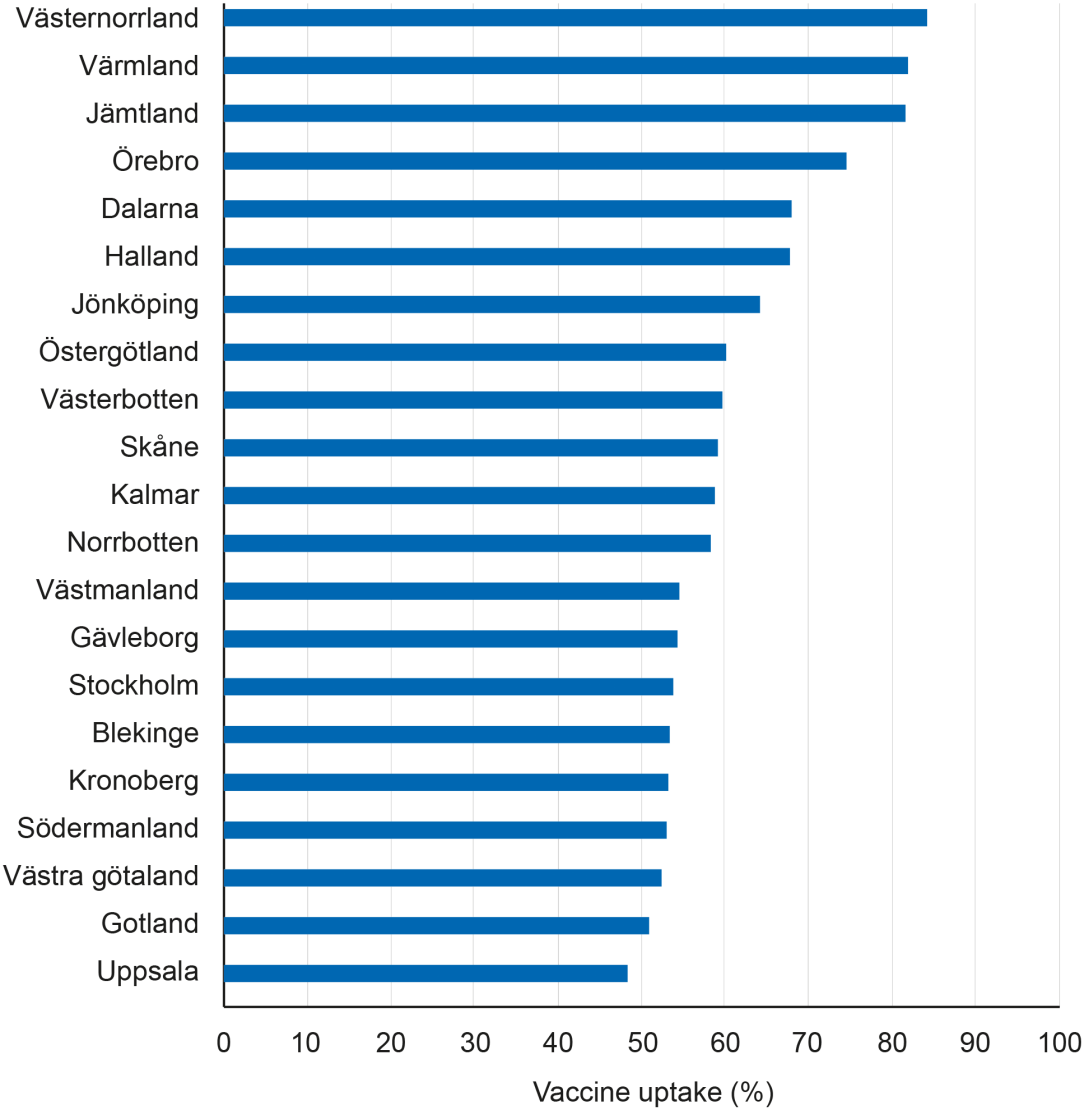
Catch-up vaccine uptake. Percentage of girls born 1993–1998 that had received at least one dose of HPV vaccine on 30 September 2014, presented by county.

By 30 September 2014, 190 289 girls born 1993–1998 (59%) had received at least one dose of HPV vaccine. The county-level catch-up vaccine uptake varied between 49–84% (Fig 2). The average vaccine uptake among counties offering vaccination in all schools and some of their schools were 77% and 57% respectively. The average uptake among counties that only offered vaccination in primary health care was 55%.

The output from the univariate and multivariable negative binomial regression models are presented in Table 2. Counties that invited girls in the catch-up group through personal letter/invitations had lower vaccine uptake compared to counties that did not (IRR: 0.7, 95% CI: 0.6–0.9), however the association was not significant in a multivariable model including all channels and settings as well as vaccine uptake for HPV in the primary target group (adjusted IRR: 0.9, 95% CI: 0.8–1.0). After adjusting for all information channels and other vaccination settings as well as vaccine uptake for HPV in the primary target group, counties offering vaccination to girls in all schools in the county and counties offering the vaccine in some of their schools, reached higher vaccine uptake compared to counties not offering the vaccine in any of their schools (all schools adjusted IRR: 1.3, 95% CI: 1.1–1.5, some schools adjusted IRR: 1.2, 95% CI: 1.1–1.3). The corresponding results in the two strata (girls attending voluntary education and those attending compulsory education) were very similar (Table 2).

Ten counties (50%) added at least one information channel or vaccination setting after the end of the first year of the campaign. Among these counties, 8 111 additional girls initiated the HPV vaccine series between 31 December 2012 and 30 September 2014, corresponding to 7% of the remaining target group after the first year. The vaccine uptake among these counties was 53% by 31 December 2012 and 55% by 30 September 2014. Among counties not adding information channels or settings after the first year (n = 11) 2 321 additional girls in the catch-up group initiated the HPV vaccine series between 31 December 2012 and 30 September 2014, corresponding to 8% of the remaining target group after 2012. The vaccine uptake among counties not adding information channels or settings after the first year was 65% on 31 December 2012 and 67% on 30 September 2014. Introducing one or more information channels or vaccination settings after 2012 did not explain county differences in vaccine uptake change between the two dates (IRR: 0.9, 95% CI: 0.5–1.7) after adjusting for the vaccine uptake on 31 December 2012.

## Discussion

County specific implementation strategies for HPV catch-up vaccination resulted in a large variation in vaccine uptake across Sweden. The highest uptake was reached by three counties with a county-wide school-based delivery. Counties offering HPV vaccination to catch-up groups in schools (both county wide and in some schools) reached statistically significantly higher vaccine uptake compared to counties that did not. No information channel, however, could explain differences in county-level vaccine uptake.

Free-of charge school-based delivery of vaccines in general is an effective way to achieve high vaccination coverage in the adolescent age group [18]. Since the introduction of HPV vaccination programmes for the primary target group, there have been many reports of the success associated with school-based delivery [19–25]. This is true also for the Swedish national vaccination programme where the uptake is above 80% [26]. Likewise, recent evaluations of implementation strategies for HPV catch-up vaccination among adolescents in Australia and Scotland has suggested that school-based campaigns are successful in terms of reaching high vaccination coverage [20, 22, 23]. Our study provides evidence that school-based delivery of HPV vaccine to the catch-up group results in high HPV vaccine uptake. Furthermore, as the Swedish implementation strategies varied across the country, we had the opportunity to compare with non-school-based strategies, and by applying quantitative methods to provide an explanation for the varying vaccine uptake between the counties. Our findings also suggest that delivery through schools is as effective in compulsory lower secondary education (7^th^ -9^th^ grade) as in voluntary upper secondary education (10^th^-12^th^ grade). Since this is an ecological study, we have no data to show the reasons for the more successful implementations. On this we can only speculate. Improved accessibility to vaccination may be one reason behind the higher uptake in counties with school-based catch-up vaccination.

The choice of information channel to inform the catch-up group about the vaccine and where to receive it did not explain differences in vaccine uptake between the counties in our study. Although other study designs may better explore the effect of communication strategies on vaccine uptake these results suggest that resources spent on information material, advertisement etc. has little or no effect on vaccine uptake without an effective delivery model in place. Furthermore, the counties with a school-based delivery model mainly used existing information channels through the school, which most likely demanded little extra resources for the county.

Our results only apply to the uptake of at least one dose of HPV vaccine; due to a large amount of anonymous data in parts of the voluntary vaccination register we were not able to retrieve further data on additional doses. The one dose uptake data may only be an indicator of the uptake of the recommended three doses, and the impact of implementation strategy could be different from that of one dose. Not registering a vaccination could either be due to denied consent from the girl (or her parents) or failure from the vaccinator. We have no reason to believe that these failures would vary between counties. However, if missing registration of vaccinations would be correlated to the implementation strategy, this might lead to an underestimation or an overestimation of the impact of the strategy. Overall, an organised school-based vaccine delivery might lead to less selection of vaccinees than an opportunistic delivery through primary health care, but this is most probably unrelated to vaccination registration.

Delivery of the HPV vaccine through a free-of-charge school-based program in Canada was suggested as being an effective method of ensuring high completion and on-time dosing: three doses were completed by 87–89 percent of 13 year old girls who initiated HPV vaccination [21]. Denmark is an example of high completion through a publically funded general practitioner based delivery model where 84 percent of 12 year old girls who initiated vaccination completed three doses [12]. In the USA, however, where HPV vaccination was delivered mainly by general practitioners with both public and private financing, the completion among 13–17 year old girls who initiated vaccination was 55 percent [27]. These studies suggest that the completion of three doses is also likely to vary between the Swedish counties with and without a county level organization of school-based delivery.

The ten counties that added at least one information channel or setting after the first year of the campaign had an overall lower vaccine uptake compared to counties with an unchanged strategy. However, the average increase in vaccine uptake after changing the strategy was at the same level as for the counties with unchanged strategies, suggesting that the adding of information channels or vaccination settings had minor effects, if any.

Our ecological study design does not allow conclusions at the individual level since there is no information on where each girl was vaccinated or through which information channels each girl was reached. Furthermore, other individual factors may be linked to vaccination status, as in Denmark for example, where the number of siblings, age, origin [11], income, unemployment and marital status of the mother [12] were found to be associated with HPV vaccination. These factors, however, could not be explored in our study due to the ecological design. Nevertheless, the ecological study design is adequate to explore the county variation in vaccine uptake at the population level, depending on e.g. delivery strategy for vaccination.

In conclusion, counties offering HPV vaccination to catch-up groups in all schools reached the highest vaccine uptake, and the choice of information channels did not explain differences in county-level vaccine uptake. While our study design does not allow conclusions on the individual level, the results together with previous reports, suggest that delivery of HPV vaccines through schools is needed to achieve a high vaccine uptake. We suggest future campaigns outside the established national vaccination programs, in other settings or for other vaccines targeting adolescents, to be implemented with an organized delivery model (e.g. in schools) to reach a high vaccine uptake.

## Supporting Information

S1 Table Implementation strategies by county. Information channels used to reach the catch-up group to inform about HPV vaccination and where to receive the vaccine, and vaccination settings by county, Sweden 2012–2014.
(PDF)
