# Supplementary material for: Highest Vaccine Uptake after School-Based Delivery - A County-Level Evaluation of the Implementation Strategies for HPV Catch-Up Vaccination in Sweden
Source: PLoS One. 2016 Mar 14;11(3):e0149857. doi: 10.1371/journal.pone.0149857 (PMC4790890; doi:10.1371/journal.pone.0149857)
Supplement: S1 Table — Information channels used to reach the catch-up group to inform about HPV vaccination and where to receive the vaccine, and vaccination settings by county, Sweden 2012–2014. (PDF) [file pone.0149857.s001.pdf]

Supporting information:

Highest vaccine uptake after school-based delivery - a county-level evaluation of the implementation strategies for HPV catch-up vaccination in Sweden

Moa Rehn, Ingrid Uhnoo, Sharon Kühlmann-Berenzon, Anders Wallensten, Pär Sparén, Eva Netterlid

S1 Table. **Implementation strategies by county.** Information channels used to reach the catch-up group to inform about HPV vaccination and where to receive the vaccine, and vaccination settings, by county, Sweden 2012-2014

	**Information channels**										**Vaccination settings**			
County	Targeted information	Smart phone app	County website	Media coverage	School-based information	Letter/ invitation	Advertisement	Social media	Cinema commercial/ YouTube	On-line health care consulting	All schools	Some schools	Other health care centre	Primary health care centre
Blekinge						X	X							X
Dalarna				X		X	X					X		X
Gotland						X	X							X
Gävleborg			X	X		X	X							X
Halland			X	X		X	X	X		X		X		X
Jämtland			X		X		X				X			X
Jönköping			X		X	X	X	X	X		X			X
Kalmar						X								X
Kronoberg				X		X								X
Norrbotten				X	X	X	X	X						X
Skåne			X	X	X	X	X	X	X	X		X	X	X
Stockholm					X	X	X	X				X		X
Södermanland		X	X		X	X	X		X					X
Uppsala		X	X	X		X	X		X				X	X
Värmland					X	X					X			X
Västerbotten	X		X			X	X		X			X		X
Västernorrland					X						X			X
Västmanland					X	X	X		X			X		X
Västra Götaland	X					X	X	X	X			X		X
Örebro				X	X	X	X					X		X
Östergötland		X	X			X	X		X					X

X= Information channel or vaccination setting implemented

Supporting information:

Highest vaccine uptake after school-based delivery - a county-level evaluation of the implementation strategies for HPV catch-up vaccination in Sweden

Moa Rehn, Ingrid Uhnoo, Sharon Kühlmann-Berenzon, Anders Wallensten, Pär Sparén, Eva Netterlid
